# Association between chinese visceral adiposity index and risk of kidney stones in a health screening population: an ultrasonography based cross-sectional study

**DOI:** 10.1186/s12882-024-03627-6

**Published:** 2024-06-11

**Authors:** XiXuan Cai, MingYing Xu, Liangli Chen, YiLin Huang, KeQing Shen, JieRu Chen, LuSha Li, Jianjiang Pan, Tao Chen, Liying Chen

**Affiliations:** 1 Department of General Practice, Sir Run Run Shaw Hospital, School of Medicine, Zhejiang University, Zhejiang, Hangzhou, 310020 China; 2 Department of Pathology, Second Affiliated Hospital of Wenzhou Medical University, Zhejiang, Wenzhou, 325027 China; 3 Jianqiao Community Health Service Center, Shangcheng District, Zhejiang, Hangzhou, 310021 China

**Keywords:** Cross-sectional study, CVAI, Kidney stones, Chinese visceral adiposity index, Ultrasonography

## Abstract

**Background:**

Obesity is an important risk factor for kidney stones(KS). Chinese Visceral Adiposity Index (CVAI), as a specific indicator for visceral obesity in the Chinese population, can more accurately assess the visceral fat content in Chinese individuals compared to Visceral Adiposity Index (VAI). However, the association between CVAI and risk for KS has not been studied.

**Methods:**

A total of 97,645 participants from a health screening cohort underwent ultrasound examinations for the diagnosis of kidney stones, along with measurements of their CVAI. Logistic regressions were utilized to determine the relationship between different quartiles of CVAI and the incidence of kidney stones. Simultaneously, subgroup analysis and the computation of dose-response curves were employed to pinpoint susceptible populations.

**Results:**

Among the participants, 2,888 individuals (3.0%) were diagnosed with kidney stones. The mean CVAI values ± standard deviation for the four groups were: Q1 (18.42 ± 19.64), Q2 (65.24 ± 10.39), Q3 (98.20 ± 9.11), and Q4 (140.40 ± 21.73). In the fully adjusted multivariable model, CVAI was positively correlated with urolithiasis (OR = 1.001; 95% CI = 1.000, 1.002). Compared with the first quartile of CVAI, the population in the fourth quartile of CVAI had a higher prevalence of kidney stones (OR = 1.231; 95% CI = 1.066, 1.415). Through subgroup analysis, a positive correlation between CVAI and the risk of kidney stones was found in non-smokers (OR = 1.001, 95%CI:1.000, 1.002), non-drinkers (OR = 1.001, 95%CI:1.000, 1.002), non-hypertensive subgroups (OR = 1.003, 95%CI:1.002, 1.003), and non-diabetes subgroups (OR = 1.001, 95%CI:1.000, 1.002).

**Conclusion:**

The findings suggest that CVAI could be a reliable and effective biomarker for assessing the potential risk of kidney stone prevalence, with significant implications for the primary prevention of kidney stones and public health.

## Introduction

Kidney stones, medically known as nephrolithiasis or urolithiasis, are a common urinary condition characterized by the formation of solid mineral deposits within the renal system, leading to severe pain, urinary tract obstruction, and various complications [1, 2].The global prevalence of kidney stones is estimated to be approximately 7.2–7.7% [3], with an incidence in China of around 5.8% [4], imposing a significant burden on healthcare systems and individuals [5]. The causes of kidney stones are multifaceted, encompassing dietary factors, genetics, fluid intake, and metabolic abnormalities [6].

Among the myriad factors drawing attention during the formation of kidney stones, the role of obesity has emerged as an intriguing research area [7, 8]. Obesity, characterized by excessive body fat accumulation, has been linked to a spectrum of metabolic disorders and systemic health issues, including cardiovascular diseases, diabetes, and metabolic syndrome [8]. The Visceral Obesity Index (VAI), a relatively recent and promising metric for quantifying visceral fat, integrates waist circumference, body mass index (BMI), and other anthropometric data to provide a comprehensive assessment of abdominal obesity [9]. Previous studies have revealed a robust association between visceral fat and metabolic disorders, such as insulin resistance, type 2 diabetes, and cardiovascular disease [10–12]. Given the intimate connection between metabolic abnormalities and kidney stone formation, VAI may serve as a valuable predictor of kidney stone risk.

While investigations have delved into the relationship between obesity and kidney stones, with a focus on VAI, which is primarily developed and validated in Caucasian populations, conclusive studies are still lacking, particularly within the Chinese population. Recent research has indicated a stronger association between the Chinese Visceral Adiposity Index (CVAI), tailored for Chinese populations, and metabolic diseases, cardiovascular diseases, and diabetes complications in Asian populations [13, 14]. Therefore, it becomes imperative to elucidate the link between quantified visceral obesity using CVAI and the incidence of kidney stones.

The primary objective of this cross-sectional study was to investigate the association between CVAI and the risk of kidney stones in healthy populations. We will employ abdominal ultrasound as a diagnostic tool to determine the presence and severity of kidney stones among participants. Through the analysis of the association between CVAI and kidney stones, we aim to provide fresh insights into the identification of risk factors for kidney stones and to offer a more precise approach for their prevention and early intervention.

## Materials and methods

### Study design and participants

The baseline clinical data included in this analysis were sourced from individuals who underwent health examinations at the Health Promotion Center of Sir Run Run Shaw Hospital, Zhejiang University, in Hangzhou, China, from January 2017 to December 2019. A total of 169,964 individuals were initially included after excluding patients with malignancies, cerebral hemorrhage, cerebral infarction, heart disease, liver dysfunction, end-stage renal disease, and autoimmune diseases. Subsequently, participants with missing baseline clinical data were excluded, leaving a final sample of 97,645 individuals.

### Outcome and exposure factor

The primary outcome measure in this study was the presence or absence of kidney stones in the subjects. Throughout the study, renal ultrasonography (UTUS) was conducted by trained radiologists using the same model of ultrasound machines from the United States, equipped with 3.0–5.0 MHz frequency transducers. Detailed records were maintained for stone size, quantity, location, degree of renal hydronephrosis, and other urinary tract abnormalities.

The main exposure factor of interest was the Chinese visceral adiposity index (CVAI), which was utilized as the primary variable. CVAI was calculated based on gender-specific mathematical models as follows: for females, CVAI=-187.32 + 1.71×age + 4.23×BMI + 1.12×WC + 39.76×lgTG-11.66×HDL-C, and for males, CVAI=-267.93 + 0.68×age + 0.03×BMI + 4.00×WC + 22.00×lgTG-16.32×HDL-C. The calculated CVAI values are presented in Table 1. CVAI primarily reflects the visceral fat content within the body, with higher CVAI values indicating greater visceral fat content and a higher predicted incidence of cardiovascular disease.

### Covariates

The medical history was systematically collected by well-trained general practitioners at Zhejiang University Affiliated Sir Run Run Shaw Hospital. It included comprehensive information such as chief complaints, current medical conditions, past medical history, personal history, family history, and physical examinations. Alcohol consumption was categorized into current drinkers (those who consumed alcohol daily for more than 6 months) and non-current drinkers. Smoking status was divided into current smokers (those who smoked daily for more than 6 months) and non-current smokers.

Measurements of body weight, height, blood pressure (BP), and waist circumference (WC) were taken by trained nurses. The Body Mass Index (BMI) was calculated as the ratio of weight (in kg) to the square of height (in m^2). Various laboratory tests were conducted, including assessments of total cholesterol (TC), triglycerides (TG), high-density lipoprotein cholesterol (HDL-C), blood urea nitrogen (BUN), serum creatinine (CR), and serum uric acid (UA). Urinalysis was performed at the hospital laboratory and included parameters such as urine pH, specific gravity, red blood cells, white blood cells, protein, bacteria, and more.

### Statistical analyses

Continuous variables are reported as mean ± standard deviation (SD), while categorical variables are presented as proportions. Analytical comparisons utilized t-tests for continuous variables and chi-square tests for categorical variables.

The association between CVAI and kidney stones was explored through logistic regression models. Model 1 remained unadjusted, while Model 2 incorporated adjustments for sex, smoking, alcohol consumption, hypertension, and diabetes. Model 3 extended these adjustments to include systolic blood pressure, diastolic blood pressure, serum uric acid (UA), glycosylated hemoglobin(HbA1c), blood urea nitrogen (BUN), serum creatinine (CR), urine specific gravity, uric pH, and urine protein, building upon Model 2.

To comprehensively investigate the relationship between CVAI and kidney stones, multivariable logistic regression was performed. CVAI was considered both as a continuous variable and categorized into four quartiles. Trends were assessed by treating CVAI quartiles as continuous variables. Additionally, we proceeded to examine if there existed a non-linear relationship between CVAI and the likelihood of kidney stones through the utilization of a generalized additive model (GAM) and curve fitting. If such a relationship was identified, we applied a two-piecewise linear regression model to determine the threshold effect of CVAI on kidney stones, based on the smoothing plot. We employed a recursive technique to automatically ascertain the inflection point, leveraging the maximum model likelihood. Subgroup analyses were conducted using hierarchical logistic regression models, encompassing all potential confounding factors outlined in the baseline table.

The statistical analyses were carried out using R version 4.0.3 software (http://www.R-project.org/) and relevant packages, including “mgcv”, “visreg”, and “broom”. A two-sided *p*-value < 0.05 was considered statistically significant, ensuring a robust evaluation of the associations observed in the study.

## Results

### Baseline characteristics of study participants

The study population characteristics at baseline were presented as in overall and quartiles of CVAI (Table 1). In total, 97,645 participants with mean age of 44.6 ± 12.0 years were enrolled in the current analysis. The mean ± SD of the CVAI in the four groups are Q1 (18.42 ± 19.64), Q2 (65.24 ± 10.39), Q3 (98.20 ± 9.11), and Q4 (140.40 ± 21.73). Table 1 shows the baseline characteristics of different CVAI groups. According to the CVAI categories, kidney stones ever accounted for1.8, 2.7, 3.4, and 3.9% in groups Q1, Q2, Q3, and Q4, respectively.

**Table 1:** Characteristics of participants divided by quartile of CVAI

Characteristic	Overall, *N* = 97,645^1^	Q1, *N* = 24,412^1^	Q2 *N* = 24,411^1^	Q3, *N* = 24,411^1^	Q4, *N* = 24,411^1^	*p*-value^2^
CVAI	80.56 ± 47.53	18.42 ± 19.64	65.24 ± 10.39	98.20 ± 9.11	140.40 ± 21.73	< 0.001
Age (years)	44.6 ± 12.0	35.1 ± 8.6	44.2 ± 10.1	48.1 ± 10.8	51.0 ± 11.6	< 0.001
BMI(kg/m2)	23.8 ± 3.3	20.4 ± 1.8	22.8 ± 1.9	24.7 ± 1.9	27.4 ± 2.6	< 0.001
Sex, n(%)						< 0.001
Female	42,683 (44%)	18,825 (77%)	12,343 (51%)	7,460 (31%)	4,055 (17%)	
Male	54,962 (56%)	5,587 (23%)	12,068 (49%)	16,951 (69%)	20,356 (83%)	
smoking, n(%)						< 0.001
Current smoker	2,054 (2.1%)	169 (0.7%)	400 (1.6%)	574 (2.4%)	911 (3.7%)	
Non-smoker	95,591 (98%)	24,243 (99%)	24,011 (98%)	23,837 (98%)	23,500 (96%)	
Alcohol consumption, n(%)						< 0.001
Current drinker	1,994 (2.0%)	136 (0.6%)	404 (1.7%)	583 (2.4%)	871 (3.6%)	
Non-drinker	95,651 (98%)	24,276 (99%)	24,007 (98%)	23,828 (98%)	23,540 (96%)	
hypertension, n(%)						< 0.001
No	84,575 (87%)	24,139 (99%)	22,890 (94%)	20,478 (84%)	17,068 (70%)	
Yes	13,070 (13%)	273 (1.1%)	1,521 (6.2%)	3,933 (16%)	7,343 (30%)	
Diabetes, n(%)						< 0.001
No	93,404 (96%)	24,277 (99%)	23,869 (98%)	23,136 (95%)	22,122 (91%)	
Yes	4,241 (4.3%)	135 (0.6%)	542 (2.2%)	1,275 (5.2%)	2,289 (9.4%)	
SBP(mmHg)	122 ± 16	111 ± 13	120 ± 15	126 ± 15	131 ± 15	< 0.001
DBP(mmHg)	73 ± 11	67 ± 9	71 ± 10	75 ± 10	79 ± 11	< 0.001
TG (mmol/L)	1.60 ± 1.40	0.84 ± 0.35	1.27 ± 0.65	1.78 ± 1.08	2.52 ± 2.13	< 0.001
TC (mmol/L)	4.82 ± 0.95	4.48 ± 0.82	4.79 ± 0.89	4.97 ± 0.94	5.03 ± 1.02	< 0.001
HDL-C (mmol/L)	1.25 ± 0.32	1.49 ± 0.32	1.30 ± 0.29	1.17 ± 0.26	1.06 ± 0.23	< 0.001
LDL-C (mmol/L)	2.73 ± 0.76	2.44 ± 0.66	2.78 ± 0.72	2.89 ± 0.77	2.82 ± 0.81	< 0.001
HbA1c	5.40 ± 0.75	5.11 ± 0.43	5.29 ± 0.57	5.48 ± 0.76	5.73 ± 0.98	< 0.001
UA (µmol/L)	351 ± 91	293 ± 69	332 ± 82	371 ± 85	406 ± 86	< 0.001
BUN (mmol/L)	5.83 ± 6.82	5.26 ± 5.69	5.82 ± 6.81	6.02 ± 7.23	6.21 ± 7.39	< 0.001
CR (µmol/L)	71 ± 16	63 ± 13	69 ± 16	74 ± 17	77 ± 16	< 0.001
Urine pH	6.25 ± 0.70	6.32 ± 0.71	6.33 ± 0.71	6.23 ± 0.69	6.13 ± 0.66	< 0.001
Urine specific gravity	1.021 ± 0.006	1.020 ± 0.007	1.020 ± 0.006	1.021 ± 0.006	1.021 ± 0.006	< 0.001
Urine protein						< 0.001
negative	71,941 (74%)	17,582 (72%)	18,295 (75%)	18,350 (75%)	17,714 (73%)	
postives	20,685 (21%)	5,540 (23%)	5,114 (21%)	4,881 (20%)	5,150 (21%)	
weakly postive	5,019 (5.1%)	1,290 (5.3%)	1,002 (4.1%)	1,180 (4.8%)	1,547 (6.3%)	
Kidney Stones						< 0.001
NO	94,757 (97%)	23,976 (98%)	23,747 (97%)	23,569 (97%)	23,465 (96%)	
Yes	2,888 (3.0%)	436 (1.8%)	664 (2.7%)	842 (3.4%)	946 (3.9%)	

^1^Mean ± SD; n (%)

^2^Kruskal-Wallis rank sum test; Pearson’s Chi-squared test

BMI, body mass index; SBP, systolic blood pressure; DBP, diastolic blood pressure; TG, triglyceride; TC, total cholesterol; HDL-C, high-density lipoprotein cholesterol; LDL-C.Low-density lipoprotein; HbA1c, glycosylated hemoglobin; UA, urine acid; BUN, blood urea nitrogen; CR, creatinine;

### Association between CVAI and kidney stones

**Fig. 1:**
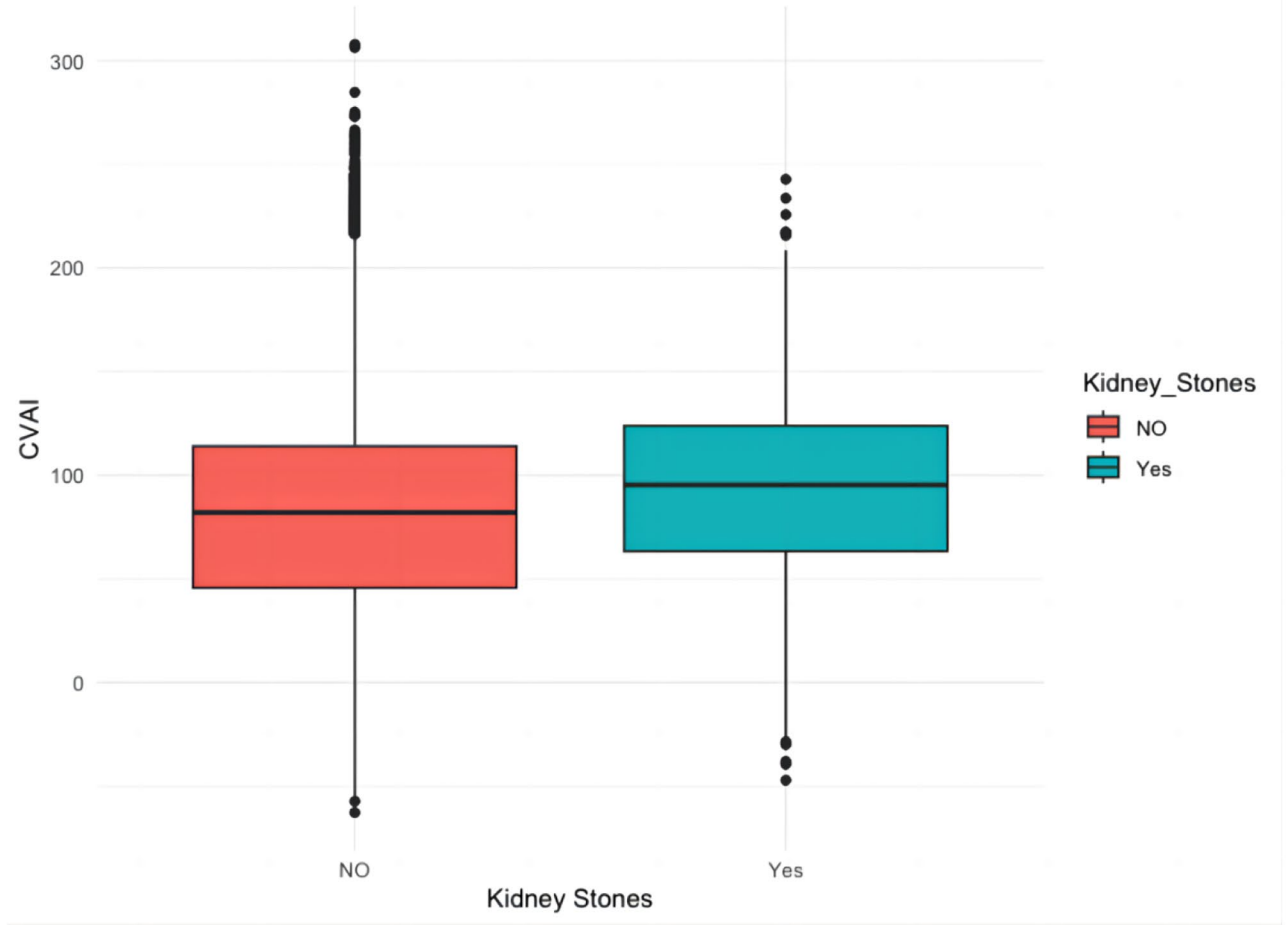
Comparison of CVAI between patients with kidney stones and non-kidney stones

Differences in CVAI between the two groups with and without kidney stones are shown in Fig. 1. Results of analysis revealed that participants with kidney stones exhibited a higher CVAI than those those without kidney stones (*p* < 0.001).

In the multivariate regression analyses, which considered different adjustments to account for confounding factors affecting the correlation, it was observed that CVAI exhibited a positive correlation with the occurrence of kidney stones in model 1 [OR (95%CI) = 1.006 (1.005, 1.007)], model 2 [1.003 (1.001, 1.004)], and model 3 [1.001 (1.000, 1.002)].Besides, compared to Q1, the participants in group Q4 had a significantly increased risk of developing kidney stones in model 1 [2.217(1.978, 2.489)], model 2 [1.474 (1.295, 1.680)], and model 3 [1.231 (1.066, 1.415)]. P for trend in all three models was less than.05 (Table 2).

**Table 2:** Association of CVAI with kidney stones

Exposure	Model 1*	Model 2†	Model 3‡
CVAI (continuous)	1.006 (1.005, 1.007) < 0.001	1.003 (1.001, 1.004) <0.00001	1.001 (1.000, 1.002) 0.031
Quartile of CVAI			
Q1	Ref	Ref	Ref
Q2	1.538(1.361, 1.738)<0.001	1.278(1.128, 1.449)<0.001	1.203(1.060, 1.368)0.004
Q3	1.965(1.749, 2.210)<0.001	1.434(1.265, 1.627)<0.001	1.268(1.112, 1.446)<0.001
Q4	2.217(1.978, 2.489)<0.001	1.474(1.295, 1.680)<0.001	1.231(1.066, 1.415)0.004
*p* for trend	< 0.001	< 0.001	0.003

*Model 1: not adjusted.

†Model 2: adjusted for sex + smoking + Alcohol consumption + hypertensive + Diabetes

‡Model 3: model 2-further adusted for SBP + DBP + UA + HbA1c + BUN + CR + Urine pH + Urine specific gravity + Urine_protein

**Fig. 2:**
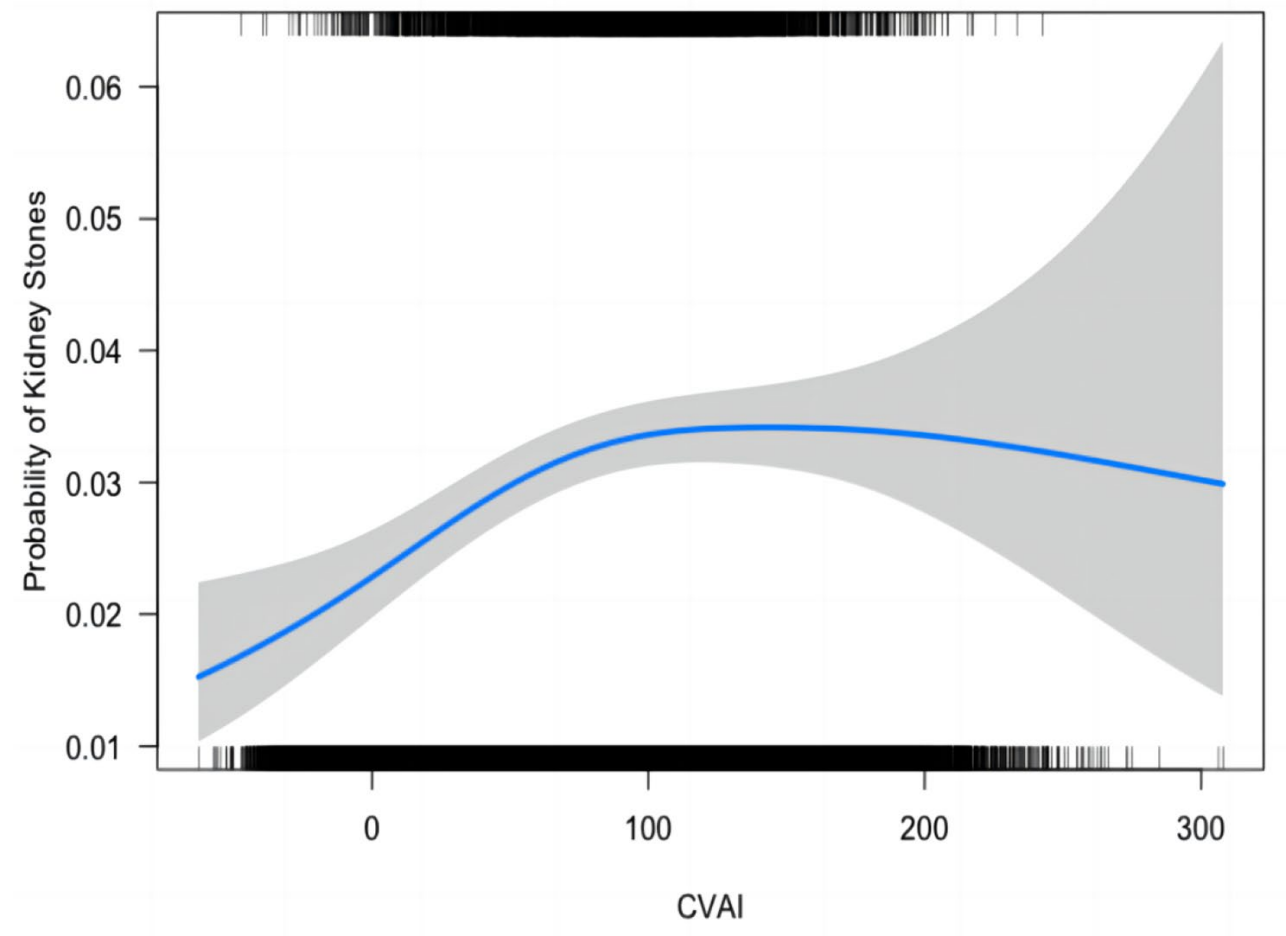
Smooth curve fitting of kidney stones and CVAI Smooth curve fitting was performed using GAM to explore the association between kidney stones and CVAI.

The relationship between CVAI and the occurrence of kidney stones was examined using Generalized Additive Model (GAM), smooth curve fitting, and piecewise linear regression techniques, as summarized in Table 3 and illustrated in Fig. 2 depicts the outcomes from the fully adjusted model, revealing a curvilinear association between CVAI and the incidence of kidney stones. The plotted data indicated that, as CVAI increased, the risk of developing nephrolithiasis followed a parabolic pattern, gradually plateauing after reaching a certain CVAI value. Subsequently, we conducted piecewise linear regression to identify the infection point (Table 3). When CVAI was < 100.00, each unit increase in CVAI corresponded to a 3‰ increase in the risk of developing kidney stones [1.003(1.001, 1.005)]. Conversely, when CVAI was > 100.00, the risk of kidney stones remained steady [1.000 (1.000, 1.001)]. The likelihood-ratio test yielded a *p*-value less than 0.05, suggesting a non-linear association between CVAI and kidney stones.

**Table 3:** Results of binary logistic regression and piecewise linear regression.*

Outcome: kidney stones	Adjusted OR (95% CI)	*p* value
Fitting by binary logistic regression model	1.001 (1.000, 1.002)	0.0311
Fitting by piecewise linear regression model		
Inflection point	100.00	
CVAI < 100.00	1.003(1.001, 1.005)	0.0002
CVAI > 100.00	1.000 (1.000, 1.001)	0.1460
Log likelihood ratio test	0.0017	

### Subgroup or interaction analyses

To assess the robustness of the CVAI-KS relationship, subgroup analyses were conducted (Table 4). We found that CVAI was positively correlated with prevalence of KS both in non-smoker (OR = 1.001, 95%CI:1.000, 1.002), Non-drinker(OR = 1.001, 95%CI:1.000, 1.002), non-hypertensive subgroups (OR = OR = 1.003, 95%CI:1.002,1.003) and non-diabetes subgroups(OR = 1.001, 95%CI:1.000, 1.002), The results showed that hypertensive have an interaction effect on the association between CVAI and KS prevalence.

**Table 4:** Subgroup analysis between the CVAI and KS prevalence

Subgroups	count	*Model1OR (95%CI)	†Model 2OR (95%CI)	‡Model 3OR (95%CI)	*p* for interaction*
Sex					0.120
Female	42,683	1.004(1.002, 1.006)	1.003(1.002, 1.005)	1.001(0.999, 1.003)	
Male	54,962	1.003(1.002, 1.004)	1.002(1.001, 1.003)	1.001(0.999, 1.002)	
Smoking					0.764
Current smoker	2054	1.002(0.997, 1.008)	1.002(0.997, 1.008)	1.000(0.994, 1.006)	
Non-smoker	95,591	1.006(1.005, 1.007)	1.003 (1.002, 1.004)	1.001 (1.000, 1.002)	
Alcohol_consumption					0.924
Current drinker	1994	1.003(0.997, 1.009)	1.004(0.997, 1.010)	1.001(0.994, 1.008)	
Non-drinker	95,651	1.006(1.005, 1.007)	1.003 (1.002, 1.004)	1.001 (1.000, 1.002)	
Hypertension					0.032
No	84,575	1.006(1.005, 1.007)	1.003 (1.002, 1.004)	1.002 (1.001, 1.003)	
Yes	13,070	1.001(0.999, 1.004)	0.999(0.997, 1.002)	0.998(0.996, 1.001)	
Diabetes					0.936
No	93,404	1.006(1.005, 1.007)	1.0013(1.002, 1.004)	1.001 (1.000, 1.002)	
Yes	4241	1.004(1.000, 1.008)	1.003(0.999, 1.008)	1.002(0.997, 1.007)	

*Model 1: not adjusted.

†Model 2: adjusted for sex + smoking + Alcohol consumption + hypertensive + Diabetes

†Model 3: adjusted for all covariates except the effect modifier

* means only in model 3.

## Discussion

Previous studies have indicated a high prevalence of obesity among individuals with kidney stones (KS) [15, 16]. However, there is still a lack of reliable obesity indices for predicting the risk of KS. By analyzing a large dataset from Run Run Shaw’s physical examination population to investigate the association between CVAI and the risk of kidney stones, we demonstrated that, after adjusting for confounding factors, a higher CVAI was associated with a greater prevalence of kidney stones, with a 1‰ increase in kidney stone risk for each additional unit of CVAI. When we divided CVAI, a continuous variable, into categorical variables based on quartiles, the incidence of KS was significantly higher in the highest CVAI group (Q4) (OR = 1.231; 95% CI = 1.066, 1.415). To date, this is the first study to investigate the relationship between CVAI and kidney stones in a healthy Chinese health screening population. Our findings suggest that an elevated CVAI is correlated with an increased risk of kidney stones, making CVAI a potentially valuable clinical indicator for assessing kidney stone risk.

CVAI, as a specific indicator for visceral obesity in the Chinese population, can more accurately assess the visceral fat content in Chinese individuals compared to VAI. Previous research has already demonstrated the significant role of CVAI in assessing various aspects of health in the Chinese population, including diabetes complications [13], liver fat deposition [17], cardiovascular diseases [18], hypertension, and stroke [19]. However, evidence regarding the association between CVAI and kidney stones (KS) is limited. Recently, a study conducted by Jiahao Wang et al [20], involving 13,871 American adults from the NHENSE database, found that for VAI values < 75.130, with each unit increase in VAI, the risk of kidney stones increased by 5‰. Another study by Bingbing Hou et al [21], using the NHENSE database and including 59,842 American adults, after standardizing VAI using LN transformation, the prevalence of kidney stones increased by 13% with each unit increase in VAI, dose-response and threshold effect analyses revealed a linear correlation between VAI and the presence of kidney stones. Some studies assessed visceral fat index (primarily involving the liver) through imaging techniques, and these studies suggested a close relationship between visceral obesity and the risk of kidney stones [22, 23]. These studies, along with our own research, suggest the potential value of CVAI in assessing the risk of kidney stones.

The formation of KS is strongly associated with low urine pH, low urine output, hyperuricemia, and increased oxalic acid excretion [24]. There are several possible mechanisms that explain the relationship between CVAI and kidney stones. First, high CVAI in patients often indicates an excessive intake of nutrients, which in turn enhances the transport of crystalline substances such as calcium, oxalate, and uric acid. Studies have found that obese stone formers tend to have higher rates of calcium, oxalate, and uric acid excretion, accompanied by lower urine pH, all of which increase the risk of kidney stones [25, 26]. Besides, a study based on American adults have showed higher VAI levels are associated with insulin resistance, which can lead to reduced ammonia secretion in the nephrons responsible for buffering H^+^ in urine by producing ammonia [27]. Studies have shown that insulin resistance can affect the transport of ammonia into the proximal lumen, leading to a reduction in ammonia content and resulting in low urine pH, which increases the risk of kidney stone formation [28]. In addition, adipose tissue is an endocrine organ that is a source of adipokines and inflammatory cytokines that can lead to insulin resistance, pro-inflammatory, prothrombotic, and hypertensive prodromal states [29]. An increase in inflammatory cytokines leads to increased oxalate absorption in the intestine and increased oxalate excretion in the urine [30].

Subgroup analysis indicated that individuals without diabetes in the highest CVAI quartile had a higher risk of developing kidney stones compared to those in the lowest quartile (Q1). However, this relationship was weaker among diabetic individuals. This implies that people with normal blood sugar levels should focus on preventing kidney stones. One possible explanation is that treatments for low blood sugar and cholesterol may affect CVAI measurements. Another possibility is that long-term high blood sugar can harm the body, leading to an increased risk of kidney stones for all diabetics, even when CVAI levels are high. In subgroup analyses, those without diabetes or hypertension consistently showed a higher risk of kidney stones. Importantly, no significant interactions were found between diabetes, hypertension, and kidney stone incidence (all interactions *P* > 0.05). This suggests that CVAI is more relevant to primary prevention rather than secondary prevention of kidney stones.

Our study has notable strengths. It’s the first to investigate the link between CVAI and kidney stone incidence in a Chinese health examination population, using ultrasound diagnosis rather than self-reported data. Despite ultrasound not being a specific marker for urinary stone disease, its utilization was chosen due to its higher accuracy compared to self-reporting. Furthermore, We meticulously controlled for confounding variables and identified a nonlinear correlation between CVAI and the incidence of kidney stones. However, our study has limitations. First, the cross-sectional design prevents us from establishing causality between CVAI and kidney stones. Despite adjusting for potential confounders, unknown variables like family history, diet, lifestyle, and medications may still influence our results. Future large-scale prospective cohort studies and randomized controlled trials (RCTs) are warranted to confirm these findings and explore causal relationships. Second, the observed incidence of kidney stones in our study population deviated from general rates(4-5.8%) [4, 31], suggesting potential bias in participant selection. As our study primarily focused on individuals undergoing health screenings in southern China, factors such as access to healthcare, socioeconomic status, and regional variations in disease prevalence may have influenced our findings. Therefore, caution is needed when generalizing our results to the broader population.

## Conclusions

The results suggest that CVAI could potentially serves as a dependable and efficient biomarker in evaluating the potential risk of kidney stone prevalence, carrying notable implications for the primary prevention of kidney stones and public health.

## Data Availability

The datasets generated and analyzed during the current study are available from the corresponding author upon reasonable request.
